# Boron Exposure Assessment of Desalinated Seawater on an Island in China

**DOI:** 10.3390/ijerph20032451

**Published:** 2023-01-30

**Authors:** Shaoxia Dong, Juexin Shi, Yuan Liu, Yingli Qu, Xin Zhao, Fengping Liu, Peng Du, Zongke Sun

**Affiliations:** Chinese Center for Disease Control and Prevention, National Institute of Environmental Health, No. 7 Panjiayuan Nanli, Chaoyang District, Beijing 100021, China

**Keywords:** boron, desalinated water, double meal intake, risk assessment

## Abstract

This study aimed to investigate the boron level in drinking water and daily boron intake of island residents, and to have a health risk assessment of the boron exposure. One-year water boron surveillance was made through the 18 selected sampling sites (5 finished water and 13 tap water) covered by 5 water treatment plants with different water sources. We recruited 220 healthy volunteers (half men and half women) from 89 families covering all age groups living in Shengshan to provide basic information and living habits. One-third of the families attended the daily food boron intake evaluation through the double meal method for three days. In each family, only one family member provided the food samples. Urine samples were collected from all subjects to get the urine boron level. Furthermore, we used the EPA model and TDI for health risk assessments. The boron level in finished water and tap water with different sources were 0.68–1.46 mg/L and 0.62–1.26 mg/L for desalinated water, 0.30–0.39 mg/L and 0.20–0.50 mg/L for reservoir water, and 0.32–0.43 mg/L and 0.20–0.79 mg/L for mixture water. The average level of water boron intake, diet boron intake, and total boron intake was 0.113 ± 0.127 mg/d, 1.562 ± 0.927 mg/d, 1.674 ± 0.939mg/d, respectively, for the select sampling subjects. There were no significant differences in total boron intake for different age groups (1.685 ± 1.216 mg/d vs. 1.669 ± 0.793 mg/d for <45 yrs vs. ≥45 yrs, *p* = 0.968) and gender groups (1.754 ± 1.009 mg/d vs. 1.633 ± 0.923 mg/d for male vs. female, *p* = 0.735). Urine boron concentrations were similar in the two age groups (1.938 mg/g creatinine vs. 1.762 mg/g creatinine for <45 yrs vs. ≥45 yrs, *p* = 0.635). There were significant differences in urinary boron between males and females (1.569 mg/g creatinine vs. 2.148 mg/g creatinine, *p* = 0.018). The largest hazard quotient (HQ) of drinking water was 0.31, and the total boron exposures in this population were 0.03 mg/kg bw per day. The study showed that there was no possible non-carcinogenic risk of water boron exposure and lower health risk of total boron exposure to humans in this region, but its toxicity should not be ignored. The subsequent studies should strengthen the analysis of the subgroup populations.

## 1. Background

Due to population growth, climate change, environmental pollution, and other factors, the shortage of freshwater resources has become a significant concern in many countries [1]. The global desalination industry has developed rapidly to alleviate the increasingly severe global water crisis and solve the problem of insufficient freshwater resources. Reverse osmosis technology is the leading technology of seawater desalination projects, and currently produces 69% of desalinated water [2]. The removal rate of boron by reverse osmosis is low, which has become a significant problem hindering the application of desalinated water in municipal water supply systems in China [3].

Boron is a widespread trace element, mainly distributed in nature in the form of borate. Boron is produced primarily by natural weathering from clay-rich sedimentary rocks. In soil, the concentration of boron is generally around 1–500 mg/kg [4]. Boron levels vary significantly in different water bodies. Compared with seawater, it is relatively low in surface water and groundwater. Notably, the data shows that boron concentration ranges from 0.1 to several 100 μg/L in precipitation, 1 to 0.5 μg/L in the lake [5], 0.03 to 2 mg/L in the river, and as high as 4 to 5 mg/L in the sea [6,7]. The distribution of boron varies in different regions. Besides water, boron is relatively high in foods such as fruits, vegetables, beans, and nuts [8,9], which is closely related to the type of plants and the growth environment.

As a trace element, boron may benefit the growth and development of humans and animals with proper intake. However, long-term exposure or excessive absorption can harm the body or lead to toxic effects [10]. It could cause acute toxicity symptoms, especially causing damage to the gastrointestinal tract, nerves, skin, and reproductive and circulatory systems when excessive boron is ingested within a short period [11,12,13]. Animal experiments have shown that boron has a certain degree of teratogenicity, and no mutagenic or carcinogenicity has been reported. Studies have also shown that boron can accumulate in the testis, inhibit cell viability, cause damage to testicular tissue, affect its function, and even cause toxic damage [14,15]. The main symptoms of the developmental toxicity of high boron always include increasing embryonic mortality, weight loss, tissue and organ damage, or malformations [16,17,18,19,20]. An epidemiological study showed that the sperm quality (such as survival viability and motility) of boron-exposed male workers decreased [21]. The spontaneous abortion and infertility rates of women whose spouses worked in boron mining areas were higher than that of the control group. Consequently, ectopic pregnancy, congenital dysplasia, and phenomena like this always exist in exposed groups. Furthermore, the chromosome Y/X ratio of sperm cells in the boron exposure group was slightly lower [13]. Nevertheless, other studies have shown that even under extreme boron exposure conditions, sperm ratio, sperm concentration, follicle-stimulating hormone and other parameters were not significantly different [22,23].

Boron exposure in the general population mainly comes from the diet and drinking. Among these exposures, diet is the most significant, accounting for about 65% of the total boron intake, with about 30% from drinking water [24]. Based on the survey and evaluation data, Rainey [25] showed that the average dietary boron intake in the United States (1989–1991), Germany (1985–1989), Mexico (1983–1986), and Kenya (1983–1986) was 1.11, 1.72, 2.12, 1.95 mg/d, and 0.89, 1.62, 1.75, 1.80 mg/d for adult men and women, respectively. Additionally, Rainey et al. [26] studied the diet of 11,009 respondents in the United States, and showed that the average daily boron intake of men, women and pregnant women was 1.17, 0.96, and 1.01 mg/d, respectively. In a New Zealand cohort study, dietary exposure to boron was 26.6–27.8 μg/kg bw/day for adult women and 26.0–27.3 μg/kg bw/day for adult men [27]. Song Xiaoyu et al. [28] investigated the boron content in grains, meat, fruits, and other foods in 12 provinces/cities of China. Their results showed that the boron content in soybeans was higher than that in other foods, and preliminarily estimated that the dietary boron intake of residents was 1.63 mg/d. The study also pointed out that due to dietary habits, geographical factors, and other reasons, there were differences in food boron level and intake amount between the investigated areas and other areas.

Boron is difficult to be removed via conventional water treatment processes, so boron in drinking water from different regions and sources varies greatly. One investigation carried out in 49 key environmental protection cities of China indicated that boron in source water ranged between 0.003 to 0.37 mg/L [29]. Research shows that it is below 0.1 mg/L in municipal water supplies of Canada [30], 0.22–1.3 mg/L in Chile [31], and 0.083–12.898 mg/L in the northwest of Iran [32]. The World Health Organization (WHO) report in 2022 pointed out that the concentration of boron in drinking water is less than 0.5 mg/L in most areas of the world [33]. In the fourth edition of the *Guidelines for Drinking-Water Quality* published in 2011, the WHO revised the guidelines of boron in drinking water from 0.5 mg/L to 2.4 mg/L based on the careful consideration of the health effects and complex reverse osmosis desalination removal process [34]. In the current standard for drinking water quality (GB 5749-2006) [35] in China, the limit of boron is 0.5 mg/L, and it has been revised to 1.0 mg/L in the new standard, which will be implemented in 2023 [36]. Based on the estimated adult daily water intake of 2L and the current limit of 0.5 mg/L, the daily limit of drinking boron intake for Chinese adults is 1.0 mg/d. Therefore, this study intended to select an area that uses desalinated water all year round to monitor the boron level in drinking water, evaluate the boron intake of residents through the double meal method, and assess the internal boron exposure by urine boron detection. Furthermore, we aimed to evaluate the health risk assessment (HRA) by water boron exposure and dietary intake while providing essential data for revising boron water quality standards and subsequent health impact assessments.

## 2. Materials and Methods

### 2.1. Location

**Figure 1:**
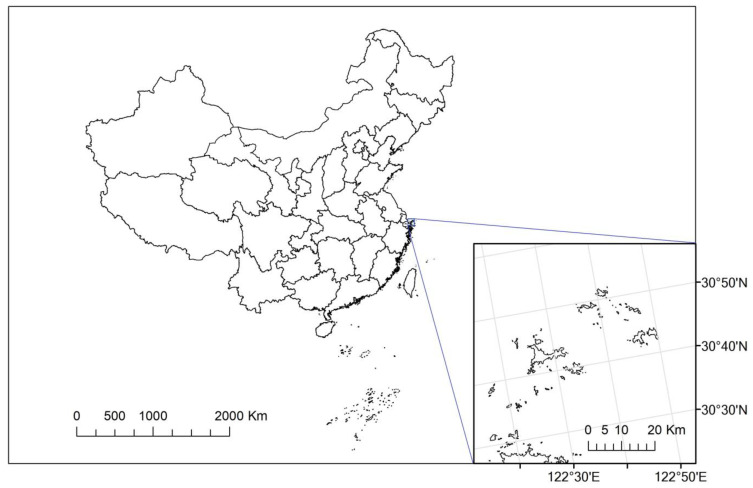
Geographic location of Shengsi County, China.

Shengsi County [37] was selected as the research site, and it is an island area under the jurisdiction of Zhoushan City, Zhejiang Province. Located to the east of Hangzhou Bay, southeast of the Yangtze River Estuary, between 30°24′ to 31°04′ north latitude and 121°30′ to 123°25′ east longitude, it is in the eastern part of Zhejiang province and the northern part of the Zhoushan archipelago (see Figure 1). It consists of 630 islands, of which 16 are inhabited islands with more than 100 permanent residents. The total land and sea area of Shengsi County is 8824 square kilometers, and nearly 99% of this consists of the sea. The county governs 3 towns and 4 townships, with a registered resident population of 72,534 as of the end of 2021. It is one of the island counties with a severe freshwater shortage in China, and the average precipitation over the years is 1105.8 mm. The lack of freshwater resources has become an essential factor restricting local economic development and the improvement in living standards. To solve this problem, a reverse osmosis seawater desalination system with a daily output of 500 tons was established in 1997, making Shengsi County the first area to use desalinated seawater as drinking water in China.

### 2.2. One-Year Water Boron Surveillance

**Figure 2:**
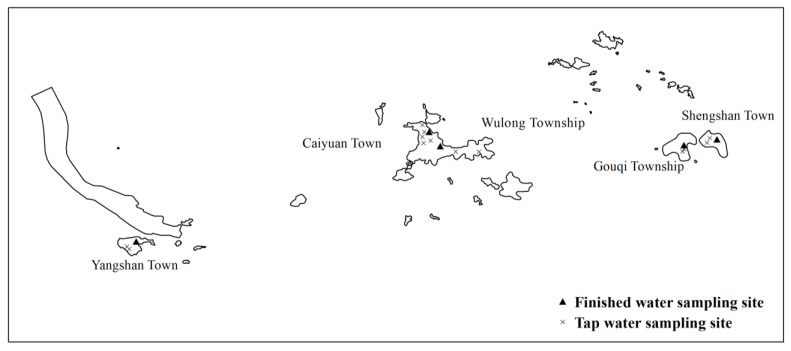
The sampling sites in Shengsi County, China.

Another group from the same team carried out water boron monitoring from May 2015 to April 2016. During the monitoring period, samples were taken 12 times in total once per month. A total of 5 finished water and 13 tap water sampling sites were set in 5 towns, including Caiyuan Town, Yangshan Town, Shengshan Town, Gouqi Township, and Wulong Township (See Figure 2). According to the different water sources, three types of water (reservoir water, desalinated water, and mixed water) were collected within the corresponding supply time. 

Water samples were collected by polypropylene containers of 50mL capacity that were previously treated with 10% nitric acid (or hydrochloric acid) for 8h and rinsed thoroughly with distilled water three times. We waited for the containers to dry by air. Water was left running for a few minutes and drained some sediment attached to the pipe prior to the taking of the sample. The water sample was not less than 2 cm away from the bottle mouth. All the samples were kept at a temperature of approximately 4–6 °C after sampling and kept away from the light before testing. Three water samples were taken at each sampling site to ensure accuracy.

The Azomethine-H Spectrophotometry analyzed boron in water according to the national standard examination methods for drinking water GB/T5750 [38]. We randomly selected 10–20% of each batch of test samples for parallel double sample determination and standard addition recovery analysis. Quality control samples and the test samples were analyzed simultaneously to ensure accuracy.

### 2.3. Boron Intake and Exposure Investigation

#### 2.3.1. Subjects and Questionnaire

This study recruited 220 healthy volunteers (half men and half women) from 89 families covering all age groups living in Shengshan Town, Shengsi County, in May 2016. Subjects were selected according to the following criteria: living in Shengsi County for at least 2 years, having no occupational exposure history to boron, having no family history of genetic diseases, and good compliance. The study protocol was approved by the ethics committee of the National Institute of Environmental Health, China CDC (No. 20139). Adult subjects or parents on behalf of their children were fully informed of the study details and provided written consent for their participation.

The questionnaire provided basic information and details of the living habits of all subjects, including their dietary structure and drinking habits. Taking the family as the unit, 32 families (about one-third of the families) were randomly selected for double meal sampling for three consecutive days. In the selected three days, one family member was asked to collect food samples, and the other family members only filled out the daily diet log to provide the eating habits and additional related information. Family members shared the same data on food boron concentration. Three-day meal records were distributed to the subjects, who recorded their daily meal consumption.

#### 2.3.2. Diet Sample Collection and Testing

The three-day double meal method was used to estimate the total boron intake. In each of the 32 selected families, only one family member was chosen to provide the double meal samples and represent the whole family. The survey lasted for 72 h on three consecutive days, one of which was Saturday or Sunday, and the other two were working days. During each meal, the participants placed duplicate amounts of their consumed food into polyethylene bags labeled by food categories, date, and subject ID. Food samples were packed and stored in a −20 °C low-temperature refrigerator until the lab testing was undertaken within one month.

ICP-MS detected food samples according to the national standard examination methods for boric acid in foods GB/T 21918-2008 [39], and the detection limit was 0.20 mg/kg. The food was digested by microwave with concentrated nitric acid and 30% hydrogen peroxide. Following digestion, we added the standard stock solution of yttrium as the internal standard, diluting it with water and making a blank control simultaneously. Finally, we introduced the series of standard solutions into the instrument atomization system adjusted to the best conditions for measurement.

#### 2.3.3. Urine Collection and Testing

Urine samples were collected from all subjects. On the third day, plastic bottles with a capacity mark were handed out for the subjects to collect 50 mL of urine the next morning. Urine samples were kept in black plastic bags, returned to the laboratory by a cold chain, and then stored at 4–6 °C for testing.

ICP-MS detected boron in the urine. The urine samples were centrifuged, and high-purity HNO_3_ and H_2_O_2_ were added to get the analyte liquor. The reagent blank, standard series, test samples, and quality control samples were detected according to the testing procedure. Urinary creatinine was also measured according to the urine determination of creatinine spectrophotometric method WS/T 97-1996 [40].

### 2.4. Risk Assessment of Boron

#### 2.4.1. Health Risk Assessment of Water Boron

In this study, the health risk of boron in drinking water was quantitatively evaluated using the HRA model recommended by the US EPA. According to the International Agency for Research on Cancer (IARC), boron is considered to be non-carcinogenic. The reference dose (RfD) for oral intake is 0.2 mg/(kg·day), according to the Environmental Protection Agency’s Integrated Risk Information System (IRIS).

The average daily dose (ADD) [41] for chronic non-carcinogenic risk assessment is calculated according to the following formula:

ADD = C × EF × ED × IR/(AT × BW)(1)

HQ = ADD/RfD(2)

here, C (in mg/L) is the concentration of chemical concentration in water, EF (in days/year) represents the annual exposure frequency, ED (in years) is the exposure period, IR (in L/days) is the oral intake rate, AT (in days) is the average time, and BW (in kg) is body weight. Furthermore, RfD is the reference oral dose and HQ is the hazard quotient. The definition of HQ is the ratio of exposure to the chemical to the reference dose of the corresponding health effect of the chemical within a certain amount of exposure time.

The average daily drinking water volume of adults in rural areas of Zhejiang province was 1.96 L/d, and the average weight of rural residents was 59.8kg [42]. The EF is 365 days per year, and the ED is 30 years. In the non-carcinogenic effect, AT is the number of days corresponding to ED, namely 10,950 days.

HQ is an indication of relative risk. HQ ≤ 1 means that the non-carcinogenic risk may be insignificant; HQ > 1 indicates that the potential risk may be significant [41].

#### 2.4.2. Calculation of Total Boron Intake in the Double Meal Study

Total boron intake evaluation was obtained through three-day diet samples detection and questionnaires, followed by calculation with the formula [43]:

(3) $$Y_{i}=\frac{C_{i}{\;\times \;X}_{i}+C_{w}\times X_{w}}{\mathrm{BW}}$$

C_i_ (in mg/kg) is the average boron concentration in three-day meals, X_i_ (kg) is the daily diet consumption, C_w_ (in mg/L) is the water boron concentration, X_w_ (in L)is the daily water consumption, and BW (in kg) is the body weight. Furthermore, Y_i_ (in mg/kg bw per day) is the daily average boron intake, which is used to evaluate the possible risk by comparing it with the tolerable daily intake(TDI). WHO recommended a TDI of 0.17 mg/kg bw per day for boron as the guideline value, which is based on the uncertainty factor and BMDL_05_ (lower confidence limit on the benchmark dose for a 0.5% response) [44].

### 2.5. Statistical Analysis

All statistical analyses were performed using SPSS 22.0. Descriptive statistics were used to present the general information of subjects. Measurement data were represented by mean ($\;\bar{X}$) and standard deviation (SD), and enumeration data were represented by a number (N) and percentage (%). Two independent sample *t*-tests was used to compare the average of two samples for normal distribution, and Mann-Whitney’s non-parametric test was used for skewed distribution. The difference was statistically significant when *p* < 0.05.

## 3. Results

### 3.1. One-Year Water Boron Surveillance

A total of 235 water samples were collected during the monitoring period from May 2015 to April 2016, of which 97 were finished water and 138 were tap water. According to the type of water source, 111 water samples belonged to reservoir water, 103 were desalinated water, and 21 were mixed water. The boron concentration in finished water and tap water from different sources is shown in Table 1. It can be seen that the boron concentration in finished water from the desalinated water was relatively high, ranging from 0.68 to 1.46 mg/L, and the boron level in the corresponding tap water was also high, ranging from 0.62 to 1.26 mg/L, which is 1.24 to 2.52 times the current limit stipulated in China’s drinking water standards, while the boron content in the tap water of the reservoir source was the lowest, with a range of 0.20 to 0.50 mg/L. Notably, reduced water boron can be obtained when desalinated seawater is mixed with reservoir water as a water source.

### 3.2. General Information of Study Subjects

A total of 220 people were surveyed, and only 199 people (93 males and 106 females) with complete information were included for analysis. The 199 participants were between 1 and 88 years of age, with an average age of 46. The average height and average weight of the total subjects were 161.47 cm and 66.65 kg, respectively. Nearly half of the population had a normal BMI, followed by overweight, obesity, and underweight. A total of 99.5% (194/195) of subjects were of Han nationality, and 69.8% (132/189) of the adults had completed junior high school or below. Furthermore, the average annual family income was about 90,000 yuan (see Table 2).

### 3.3. Total Boron Intake

In total, 288 meal samples were obtained for 32 family representatives with three meals a day for 3 days. The average boron in water boron intake, diet boron intake, and total boron intake was 0.113 ± 0.127 mg/d, 1.562 ± 0.927 mg/d, and 1.674 ± 0.939 mg/d, respectively (Table 3). Boron intake by drinking water of the group over 45 years old was higher than that of the under 45 years old group (0.136 ± 0.130 mg/d vs. 0.067 ± 0.088 mg/d, *t* = −1.489, *p* = 0.147), while diet boron intake was a little lower than that of the under 45 years old group with no significant difference. The average boron intake of males through water, diet and the combination of both was slightly higher than that of females (0.115 ± 0.178 mg/d vs. 0.111 ± 0.096 mg/d, *t* = 0.067, *p* = 0.948; 1.639 ± 1.038 mg/d vs. 1.521 ± 0.888 mg/d, *t* = 0.336, *p* = 0.739; 1.754 ± 1.009 mg/d vs. 1.633 ± 0.923 mg/d, *t* = 0.342, *p* = 0.735).

### 3.4. Boron in Urine

The average urine boron corrected by creatinine in 199 subjects was 1.851 mg/g creatinine. As is shown in Table 4, there was no statistical difference in urinary boron between different age groups (1.938 mg/g creatinine vs. 1.762 mg/g creatinine, *p* = 0.635). However, urine boron corrected by creatinine was significantly higher in females than in males (2.148 mg/g creatinine vs. 1.569 mg/g creatinine, *p* = 0.018).

### 3.5. Risk Assessment

Results showed that the HQs of different types of water samples were less than 1, and the drinking water possessed no carcinogenic risk. The minimum HQ identified was from the tap water from the reservoir source and from the mixture of water from the reservoir and desalinated water (HQ = 0.03 for both). The maximum HQ identified was from the finished water with a desalinated seawater source (HQ = 0.24) (See Table 5).

The average total boron intake was 0.03 mg/kg bw per day, which was far lower than the TDI (0.03 mg/kg bw per day vs. 0.17 mg/kg bw per day, *t* = −65.819, *p*<0.001). Additionally, there was no significant difference between age groups (0.033 ± 0.013 mg/kg bw per day vs. 0.028 ± 0.011 mg/kg bw per day, *t* = 1.298, *p* = 0.204) or between males and females (0.029 ± 0.011 mg/kg bw per day vs. 0.030 ± 0.013 mg/kg bw per day, *t* = −0.218, *p* = 0.829) (Table 6).

## 4. Discussion

Generally, boron concentration in drinking water is below 0.5mg/L depending on the source difference [34]. With more and more desalinated water being adopted into the water supply, WHO revised the guideline value of boron in drinking water from 0.5 mg/L to 2.4 mg/L in 2011 based on the careful consideration of health effects and the complexity of the reverse osmosis desalination process [45]. However, the global limit value of boron is not entirely consistent. A maximum acceptable concentration (MAC) of 2 mg/L is proposed for boron in Canada’s drinking water [30]. The RfD of boron is 0.2 mg/kg/day in the US [46]. In China, the current limit of boron in drinking water is 0.5 mg/L, and the revised new limit (1.0 mg/L) will be implemented in April 2023. The shortage of freshwater sources is a serious problem, and desalination is the most effective way to solve it. Currently, there are no unified water standards and health advisories for desalinated seawater. In China, boron in desalinated seawater produced by reverse osmosis generally exceeds the standard limit of current drinking water guidelines, which hinders the large-scale application of desalinated seawater into the municipal water supply system. Therefore, evaluating the boron concentration in desalinated seawater and its potential health risk will be particularly meaningful. The measurement of boron intake provides the baseline data for health effect evaluation. Therefore, this study will substantially contribute to verifying and re-evaluating boron limits in drinking water, especially for desalinated water. It will also provide additional data to contribute to the ongoing international debate on which boron intake level is safe for humans.

Generally, the boron level in desalinated water resulting from reverse osmosis is relatively high because of the higher boron level in the ocean and the lower filtration efficiency of small molecules. In this study, one-year water quality monitoring showed that drinking water boron is 0.62–1.46 mg/L, similar to the previous results of 0.72–1.6 mg/L [47]. Notably, both are higher than the current national standard limit of 0.5 mg/L and slightly higher than the forthcoming revised standard [33]. In the course of our research, we found that during the three-day double meal recording, three types of water samples from well water, tap water, and spring water were also collected, and the boron level was 0.0045 mg/L, 0.22 mg/L, 0.07 mg/L, respectively, which were lower than 0.5 mg/L. These findings are consistent with the one-year water quality monitoring results, and both indicate that boron levels can be reduced after mixing desalted seawater to meet the national standard limit requirements. Following desalination, additional boron removal measures will increase the cost of desalination, which will restrict the application of desalinated water. Therefore, a mixed water supply or innovations in desalination technology are critically needed to balance necessity and economics.

The dietary boron intake in this study is consistent with that of other countries. Our study showed that the daily boron exposure of residents of this island area is 1.674 mg/d, which is similar to the current domestic result (1.63 mg/d) reported by Song Xiaoyu [28] and some foreign literature (1.33 mg/d in Canada [48], 1.77 mg/d in Fenland [49] and 1.5 mg/d in the US [50]). In addition, the boron intakes of males are higher than that of females, in good agreement with other studies [51] (see Table 7). Data from the US [52,53] illustrated that dietary boron intake increased significantly with age (Table 8). Rahman [54] et al. have shown that the boron intake at different ages was in the order of teenagers (6 to < 16 years) > adults (≥16 years) > infants (0 to 2 years). In this study, the subjects were mainly middle-aged and older adults. Therefore, we divided them into two groups according to the age of 45. In the <45 group, total and dietary boron intake was higher than that in the ≥45 group, while water boron intake was lower. One of the possible reasons was that the elderly group drank more water (900 mL vs. 713 mL, ≥45 vs. <45) which resulted in higher boron intake from water. However, we did not divide the data into more specific groups as in the above US study because of the small sample size. Due to different consumption of water and food, an in-depth investigation is needed to explore the boron intake and related factors in different ages.

Considering that approximately 90% of boron is eliminated through urine and internal exposure indicators could be used to reflect the multi-exposure level, we collected urine samples from the surveyed population and determined the corresponding urine boron concentration. There were no relationships between age and urinary boron. However, significant differences in urine boron existed between males and females. When subjects were given a boron-rich diet [9], boron concentration in serum, saliva, and urine increased by 1.3, 1.7, and 6.0 times, respectively. Notably, the change in urinary boron was significantly more significant than blood boron. According to another multiple linear regression study [55], urinary boron had no meaningful relationship with age and gender. However, the details have yet to be confirmed.

Whether for finished or tap water, the chronic non-carcinogenic risk of boron with a desalinated water source was higher than that of the other two sources: a reservoir or a mixture water source. Importantly, the HQ of the latter two sources is very close. Furthermore, we can easily find that tap water was lower than finished water. It indicated that a mixture of water could reduce the non-carcinogenic risk of boron in desalinated water. Therefore, we can decrease the risk of desalinated water by combining it with reservoir water. However, through pipelines, the range of HQ in desalinated water became smaller, and the range in the reservoir and mixture water widened. One of the possible reasons is that borate combined with metal hydroxide in an aqueous solution precipitated in the process of transportation and reduced the risk of water boron. Although we discussed the three water types and pipes, HQs are less than 1, which illustrates that there is no non-carcinogenic risk based on existing standards. Total boron exposure results reversed when compared to intake. Furthermore, females are at greater risk of higher boron exposure than males. This suggests that we should pay attention to weight standardization in selecting risk assessment indicators in the future. Collectively, none of the total boron exposure exceeded recommended values. The highest boron intake was 0.03 mg/kg bw per day, accounting for 17.6% of the TDI. Therefore, it can be concluded that total boron exposure in the investigated population posed no risk.

## 5. Conclusions

The boron in desalinated water prepared by reverse osmosis is relatively high, which usually exceeds the current hygienic standard of drinking water. It is worthy of note that appropriately mixing a certain amount of reservoir water could be considered to reduce the boron concentration in water and stay within hygienic standards. Subsequent attention should be paid to the mixing ratio of two kinds of water so as to find the optimal proportion of human intake of trace elements. Furthermore, we have found that boron intake in different ages and genders was not significant by investigating residents in a selected island area. However, urinary boron was significantly different between males and females. No significant health risk from boron exposure has been found in this region. We need to discover the underlying mechanism in different populations. It is apparent that long-term monitoring is still recommended and required.

## Figures and Tables

**Table 1:** Boron concentrations in different types of water (mg/L).

Water Types	Desalinated Water		Reservoir		Mixture Water	
	n	Range	n	Range	n	Range
Finished water	51	0.68–1.46 *	39	0.30–0.39	7	0.32–0.43
Tap water	60	0.62–1.26 *	64	0.20–0.50	14	0.20–0.79 *

*: The boron concentration > 0.5 mg/L (the limit value).

**Table 2:** Basic information of study subjects.

Variable	Male *	Female *	Total *
*Age (yrs)*			
<45	37 (39.8%)	52 (49.1%)	89 (44.7%)
≥45	56 (60.2%)	54 (50.9%)	110 (55.3%)
*BMI*			
Underweight	5 (5.4%)	12 (11.3%)	17 (8.5%)
Normal	39 (41.9%)	57 (53.8%)	96 (48.2%)
Overweight	33 (35.5%)	20 (18.9%)	53 (26.6%)
Obesity	16 (17.2%)	17 (16.0%)	33 (16.6%)
*Nation*			
Han nationality	91 (98.9%)	103 (100.0%)	194 (99.5%)
Other	1 (1.1%)	0 (0.0%)	1 (0.5%)
*Education*			
Junior high school and below	68 (77.3%)	64 (63.4%)	132 (69.8%)
High school and above	20 (22.7%)	37 (36.6%)	57 (30.2%)
*Economic status* *(×10^4^ RMB)*	9.76 ± 10.63	8.46 ± 4.10	9.05 ± 7.77

*: Values of Economic status (×10^4^ RMB) represent $\bar{X}\pm SD$, other values represent N (%).

**Table 3:** Boron intake in different populations (mg/d).

Group	N	Water Boron Intake			Diet Boron Intake			Total Boron Intake		
		$\bar{X}\pm SD$	*t*	*p*	$\bar{X}\pm SD$	*t*	*p*	$\bar{X}\pm SD$	*t*	*p*
*Age*										
<45	11	0.067 ± 0.088	−1.489	0.147	1.618 ± 1.180	0.245	0.808	1.685 ± 1.216	0.041	0.968
≥45	21	0.136 ± 0.130			1.532 ± 0.796			1.669 ± 0.793		
*Gender*										
Male	11	0.115 ± 0.178	0.067	0.948	1.639 ± 1.038	0.336	0.739	1.754 ± 1.009	0.342	0.735
Female	21	0.111 ± 0.096			1.521 ± 0.888			1.633 ± 0.923		
*Total*	32	0.113 ± 0.127			1.562 ± 0.927			1.674 ± 0.939		

**Table 4:** Urine boron concentrations in different ages and genders (mg/g creatinine).

Group	N	Urine Boron Corrected by Creatinine		Mann-Whitney U	*p*
		Median	IQR		
*Age*					
<45	89	1.938	1.906	4703	0.635
≥45	110	1.762	1.623		
*Gender*					
Male	93	1.569	1.443	3974	0.018 *
Female	106	2.148	1.909		

*: *p* < 0.05.

**Table 5:** Chronic non-carcinogenic risk of boron exposure in 2015–2016 (HQ).

Type of Water Sample	HQ	
	Min	Max
*Finished water*		
Desalinated water	0.11	0.24
Reservoir	0.05	0.06
Mixture water	0.05	0.07
*Tap water*		
Desalinated water	0.10	0.21
Reservoir	0.03	0.08
Mixture water	0.03	0.13

**Table 6:** Total boron exposure in different populations (mg/kg bw per day).

Group	N	Total Boron Exposure	*t*	*p*
*Age*				
<45	11	0.033 ± 0.013	1.298	0.204
≥45	21	0.028 ± 0.011		
*Gender*				
Male	11	0.029 ± 0.011	−0.218	0.829
Female	21	0.030 ± 0.013		
*Total*	32	0.030 ± 0.012	−65.819	<0.001 *

* Compared with TDI by one sample *t*-test.

**Table 7:** Diet boron intake in different countries (mg B/d).

Country	Male	Female
US	1.11 ± 0.69	0.89 ± 0.57
German	1.72 ± 0.47	1.62 ± 0.76
Mexico	2.12 ± 0.69	1.75 ± 0.48
Kenya	1.95 ± 0.57	1.80 ± 0.49
Egypt	1.31 ± 0.50	1.24 ± 0.40
This research	1.64 ± 1.04	1.52 ± 0.89

**Table 8:** Dietary boron intake for different ages in the US (mg B/d).

Age	Sex	CSF II	NHANES III
7–11 month	Male/female	0.99 ± 0.12	
1–3 years	Male/female	0.86 ± 0.02	
4–8 years	Male/female	0.80 ± 0.01	
9–13 years	Male	0.90 ± 0.03	0.92 ± 0.03
	Female	0.83 ± 0.03	0.84 ± 0.03
14–18	Male	1.02 ± 0.04	1.06 ± 0.05
	Female	0.78 ± 0.04	0.80 ± 0.03
19–30	Male	1.15 ± 0.03	1.29 ± 0.04
	Female	0.87 ± 0.03	0.92 ± 0.03
31–50	Male	1.33 ± 0.03	1.42 ± 0.04
	Female	1.00 ± 0.02	1.19 ± 0.06
51–70	Male	1.34/0.02	1.42 ± 0.04
	Female	1.11/0.02	1.16 ± 0.03
70+	Male	1.25/0.03	1.28 ± 0.04
	Female	0.98/0.03	1.03 ± 0.02

## Data Availability

The datasets used and analyzed in this study are available from the corresponding author upon reasonable request.
